# Mesenteric lymph drainage alleviates hemorrhagic shock-induced spleen injury and inflammation^1^

**DOI:** 10.1590/s0102-865020190090000003

**Published:** 2019-11-25

**Authors:** Hong Zhang, Jia-yi Zhai, Hui-bo Du, Li-min Zhang, Lin-feng Li, An-qi Bian, Li-na Jiang, Zi-gang Zhao

**Affiliations:** I MB, Institute of Microcirculation, Hebei North University, Zhangjiakou, China. Acquisition, analysis and interpretation of data; technical procedures; statistics analysis; histopathological examinations; manuscript writing.; II MB, Institute of Microcirculation, Hebei North University, Zhangjiakou, China. Acquisition of data, technical procedures, statistics analysis.; III MB, Institute of Microcirculation, Hebei North University, Zhangjiakou, China. Technical procedures.; IV PhD, Institute of Microcirculation, Hebei North University, Zhangjiakou, China. Conception and design of the study, analysis and interpretation of data, technical procedures, critical revision.; V PhD, Institute of Microcirculation, Hebei North University, Zhangjiakou, China. Conception and design of the study, analysis and interpretation of data, manuscript preparation, critical revision, final approval.

**Keywords:** Shock, Hemorrhagic, Spleen, Drainage, Inflammation, Mice

## Abstract

**Purpose:**

To investigate the effect of mesenteric lymph drainage on the spleen injury and the expressions of inflammatory cytokines in splenic tissue in mice following hemorrhagic shock.

**Methods:**

Male C57 mice were randomly divided into the sham shock, shock and shock+drainage groups. The mice in both shock and shock+drainage groups suffered femoral artery bleeding, maintained mean arterial pressure (MAP) of 40±2 mmHg for 90 min, and were resuscitated. And mesenteric lymph drainage was performed in the shock+drainage group at the time of resuscitation. After three hours of resuscitation, the splenic tissues were harvested for the histological observation and protein and mRNA expression analysis of cytokines.

**Results:**

The spleen in the shock group revealed a significantly structural damage and increased mRNA expressions of MyD88 and TRAF6 and protein expressions of TIPE2, MyD88, TRIF and TRAF3 compared to the sham group. By contrast, the splenic pathological injury in the shock+drainage group was alleviated significantly, and the mRNA and protein expressions of TIPE2, MyD88, TRIF, TRAF3 and TRAF6 were significantly lower than those in the shock group.

**Conclusion:**

These results indicate that post-hemorrhagic shock mesenteric lymph drainage alleviates hemorrhagic shock-induced spleen injury and the expressions of inflammatory cytokines.

## Introduction

Hemorrhagic shock is a common clinical intensive illness and is an important cause of trauma leading to death^1^ ^–^ ^3^. The return of post-hemorrhagic shock mesenteric lymph (PHSML) is a key reason of multiple organ dysfunction caused by hemorrhagic shock. Therefore, mesenteric lymph drainage or mesenteric lymph duct ligation improve the multiple organ functions and reduce the tissue injury following hemorrhagic shock, such as lung, heart and kidney^4^ ^–^ ^10^. Recently, the role of PHSML in the pathogenesis of hemorrhagic shock has attracted increasing attention^11^ ^–^ ^13^. Hemorrhagic shock causes the immediate activation of immune system and rapid onset of inflammatory reaction, which leads to immune dysfunction and injury of immune organs^14^. Researches show that decreasing PHSML reflux is beneficial for reducing injury of splenic tissue after hemorrhagic shock^15^ ^,^ ^16^, which is mediated by TLR4/TLR2^16^. Tumor necrosis factor-alpha-induced protein 8-like 2 (TIPE2) is required for maintaining immune homeostasis, and is preferentially expressed in lymphoid tissues^17^, but the role of TIPE2 in the development of hemorrhagic shock-induced immune disorder remains unclear. In order to reveal the role of PHSML in hemorrhagic shock-induced spleen injury and inflammatory response, the present study observed the effects of PHSML drainage on the splenic tissue structure and the expressions of inflammatory cytokines, including TIPE2 and the downstream molecules of TLR4/TLR2.

## Methods

### Animals and the experimental group

Healthy and male C57 (8-10 weeks) mice purchased from Sibeifu (Beijing Biotechnology Co., Ltd.) were raised in the Animal Room and ate and drank freely. All animals used in this study were fasted for 8 hours and drank freely before the experiment. Eighteen mice were randomly divided into 3 groups (n=6 each group): sham group (anesthesia and operation, no bloodletting, sampling at the corresponding time point), shock group (established hemorrhagic shock model), shock + drainage group (established hemorrhagic shock model with drainage of mesenteric lymph). All animal experiment was performed according to the guideline and requirements of animal ethics.

### Hemorrhagic shock model

Mice were anesthetized with isoflurane (RWD Life Science Co., 217180801) and intramuscular injection of 1% pentobarbital sodium (0.07 mL/kg, Merk, P11011). Bilateral femoral arteries were separated under stereoscopic microscope and inserted into bilateral femoral arteries according to the routine method in our lab^7^. Mouse mean arterial pressure (MAP) was monitored in the left femoral artery using the PowerLab 15T four-channel multi-purpose data acquisition and analysis system (AD Instruments, Australia), and the right side was connected with 1 mL syringe and placed in NE-1000 programmed microinjection pump (American New Times Company) for bloodletting and liquid resuscitation. After 30 min-equilibrium period, bleeding was performed and MAP was maintained at the level of 40±2 mmHg for 90 min. During this period, close attention was paid to the MAP and the state of mice. After 90 min, the whole blood and equal ringer's solution were used to resuscitate at a uniform speed within 30 min. After resuscitation, all mice were placed on the operating table in supine position, and the status of MAP was closely observed for 3 hours. After 3 hours, mice were sacrificed by spinal cord dislocation method, and relevant samples were collected. After resuscitation, the mesenteric lymph was drained for 3 hours by one-time-using blood collection needle and blood vessels collection. The spleen samples were harvested from all mice for further study.

### Histopathology observation

Small pieces of spleen were fixed in 4% paraformaldehyde. Specimens were then routinely dehydrated, wax immersed and embedded, etc., in continuous sections (3 μm), including exhibition piece and fishing piece, and sealed with neutral gum; then, the splenic morphology was observed. The degree of splenic injury was evaluated with a semiquantitative scoring system^18^ ^–^ ^20^. The details were as follows: 0, the morphology of spleen white pulp was obvious, which was characterized by obvious per arterial lymphoid sheath, gerontology center, capsule zone and marginal zone; 1, mild disorder of spleen white pulp, characterized by local hyperplasia; 2, moderate disorder of spleen white pulp, blurring of the boundary between white pulp and red pulp; 3, high disorder of spleen white pulp, almost no significant difference between white pulp and red pulp.

### Real-time RT-PCR analysis

Total RNA was extracted from splenic tissue with Trizol reagent. RNA was reversely transcribed into cDNA using FastQuant RT Kit (Beijing Tiangen biology Co., Ltd.). RT-PCR was used to analyze the mRNA levels of TIPE2, MyD88, TRIF, TRAF3 and TRAF6 in splenic tissue with SYBR Green kit (Beijing Tiangen biology Co., Ltd.). PCR of GAPDH was used as an internal control with the same condition. The primers used in the experiment were synthesized by Shanghai Bioengineering Biology (Shanghai, China). The primer sequence of each gene is as follows in Table 1.

**Table 1:** Primer's sequence.

Gene	Primer's sequence
TIPE2	Forward: 5'-CGATTTCGTCAGAAGCTACG-3'
	Reverse: 5'-GGGTCAGAGTAGTGATCAAACA-3'
MyD88	Forward: 5'-CGGAACTTTTCGATGCCTTTAT-3'
	Reverse: 5'-CACACACAACTTAAGCCGATAG-3'
TRIF	Forward: 5'- CCTGTCAGCACGTTTTCTGTA-3'
	Reverse: 5'- CCACGACATAGGGGACAATGTA-3'
TRAF3	Forward: 5'- CAGCCTAACCCACCCCTAAAG-3'
	Reverse: 5'- TCTTCCACCGTCTTCACAAAC-3'
TRAF6	Forward: 5'-GAAAATCAACTGTTTCCCGACA-3'
	Reverse: 5'-ACTTGATGATCCTCGAGATGTC-3'
GAPDH	Forward: BN62304-001
	Reverse: BN62304-002

### Western blot analysis

After the protein quantification with BCA kits, the protein levels of TIPE2, MyD88, TRIF, TRAF3 and TRAF6 in splenic tissue from different groups were analyzed by SDS-PAGE electrophoresis. The protein was then transferred to polyvinylidene fluoride membrane after electrophoresis. The membranes were blocked with 5% skim milk diluted in TBST, followed by an overnight incubation with primary antibodies against β-actin (1:2000 dilution; Applygen), TIPE2 (1:1000 dilution; protein tech), MyD88 (1:1000 dilution; cell signaling technology), TRIF (1:1000 dilution; Bioworld), TRAF3 (1:1000 dilution; Bioworld), TRAF6 (1:1000 dilution; Bioworld). The membranes were subsequently incubated with a horseradish peroxidase-conjugated secondary antibody (1:5000 dilution; Applygen). Finally, images were examined with an ImageQuant LAS 4000 imager, and strip density was analyzed by Quantity One software.

### Statistical analysis

All data are present as means±standard deviation (SD) or means±standard error (SE). Difference between groups was analyzed using a one-way ANOVA with an LSD multiple-comparison test or a Student's *t*-test using SPSS software 22.0. *P*<0.05 was considered statistically significant.

## Results

### PHSML drainage alleviated the spleen injury induced by hemorrhagic shock

**Figure 1:**
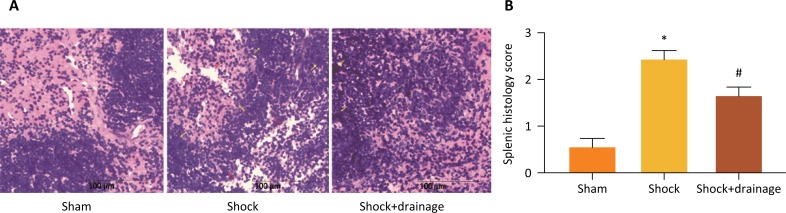
Mesenteric lymph drainage reduced hemorrhagic shock-induced spleen injury. **A.** The changes of splenic morphology in different groups (HE staining, ×200). No histological alterations were observed in the spleen obtained from the sham mice. The structural looseness and local proliferation were observed in the spleen of the shock group, which was alleviated by mesenteric lymph drainage. **B.** The splenic histological score of different groups. Data are presented as mean ± SE (n=3). *P<0.05, *vs*. the *sham* group, # *P*<0.05, *vs*. the shock group.

As shown in Figure 1, there were no significant pathological changes in splenic tissue from sham group, with the evidences of clearly lymphoid nodules and “germinal centers”. In contrast, there was serious spleen injury with the characterizations of thinner spleen cord and dilated spleen sinus in the shock group. However, there was slight spleen injury in the shock+drainage group. The results of semiquantitative score showed that hemorrhagic shock significantly increased the splenic histological score when compared to the sham group, and PHSML drainage significantly decreased the splenic histology score of the shocked mice (Fig. 1B).

### PHSML drainage decreased the mRNA expressions of TIPE2, MyD88, TRIF, TRAF3 and TRAF6 in spleen after hemorrhagic shock

**Figure 2:**
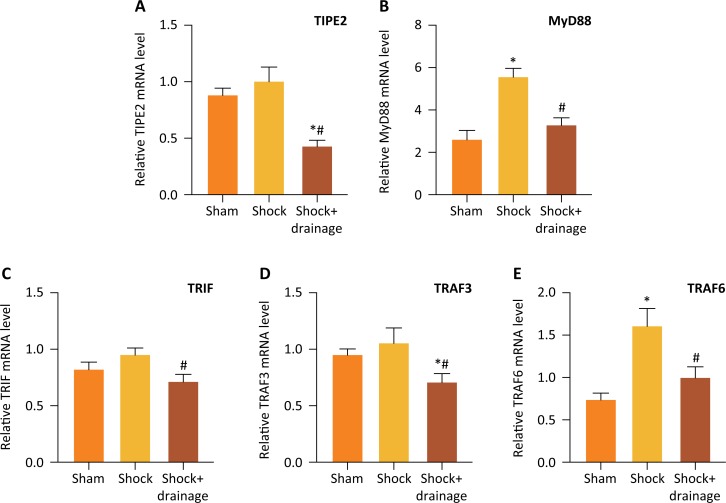
The mRNA expressions of TIPE2, MyD88, TRIF, TRAF3 and TRAF6 in murine splenic tissue following hemorrhagic shock. Compared to the sham group, hemorrhagic shock increased the level of MyD88 and TRAF6 **(B, E)** and mesenteric lymph drainage reduced the mRNA expressions of TIPE2, MyD88, TRIF, TRAF3 and TRAF6 **(A-E)**. Data are presented as mean ± SE (n=3). * *P*<0.05, *vs*. the sham group, # *P*<0.05, *vs*. *t*he shock group.

RT-PCR method was used to detect the mRNA levels of TIPE2, MyD88, TRIF, TRAF3 and TRAF6 in spleen tissue of mice following hemorrhagic shock. The results showed that hemorrhagic shock significantly increased the mRNA levels of MyD88 and TRAF6 (*P<0.05*), and slightly increased the mRNA levels of TIPE2, TRIF and TRAF3 that were no statistically difference (Fig. 2). However, PHSML drainage significantly decreased the levels of TIPE2, MyD88, TRIF, TRAF3 and TRAF6 compared with the shock group (*P<0.05*).

### PHSML drainage reduced the protein expressions of TIPE2, MyD88, TRIF, TRAF3 and TRAF6 in spleen after hemorrhagic shock

**Figure 3:**
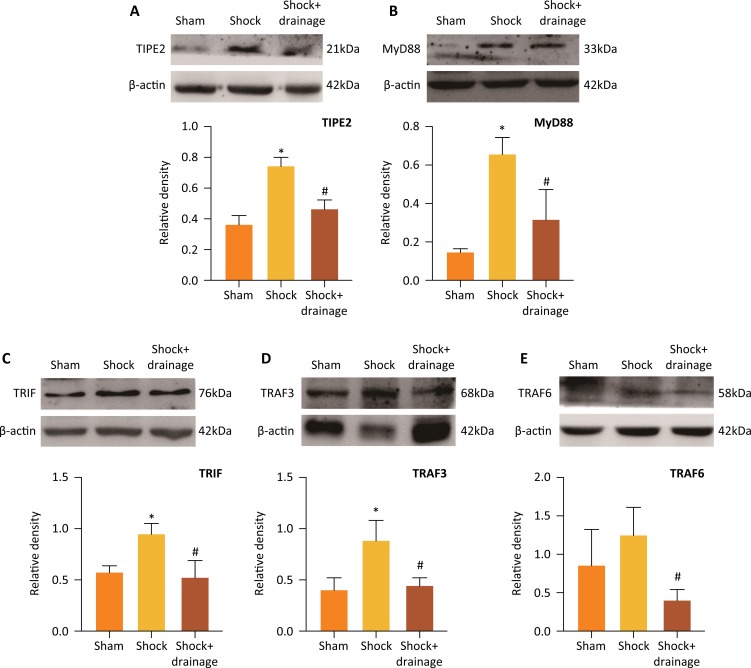
The protein expressions of TIPE2, MyD88, TRIF, TRAF3 and TRAF6 in murine splenic tissue of mice following hemorrhagic shock. Compared to the sham group, hemorrhagic shock increased the expressions of TIPE2, MyD88, TRIF and TRAF3 **(A-D)**, and mesenteric lymph drainage reduced the expressions of TIPE2, MyD88, TRIF, TRAF3 and TRAF6 **(A-E)**. Data are presented as mean ± SD (n=3). * *P*<0.05, *vs.* the sham group, # *P*<0.05, *vs. t*he shock group.

The protein expressions of TIPE2, MyD88, TRIF, TRAF3 and TRAF6 in splenic tissue of mice following hemorrhagic shock were further detected by Western blot. As shown in Figure 3, hemorrhagic shock significantly increased the protein levels of TIPE2, MyD88, TRIF and TRAF3 than those in the sham group (*P<0.05*), and slightly increased the levels of TRAF6 with no statistical difference. Compared to shock group, drainage of PHSML significantly decreased the protein levels of TIPE2, MyD88, TRIF and TRAF3 (*P<0.05*).

## Discussion

The present study found that hemorrhagic shock results in the spleen injury and enhances the protein or mRNA expressions of cytokines, such as TIPE2, TRIF, TRAF3, TRAF6 and MyD88 that are associated with inflammation and immune function. By contrast, PHSML drainage alleviates the tissue injury and decreases the expression of these cytokines in murine spleen following hemorrhagic shock.

Immune dysfunction plays a key role in the occurrence and development of multiple organ failure following hemorrhagic shock^21^. The spleen is one of the most important immune organs playing a key role in the innate and adaptive immune responses, which are frequently affected in infectious diseases^22^, and spleen injury was involved in the pathophysiology of hemorrhagic shock-induced immune dysfunction^23^. Therefore, the current study observed the change of splenic histopathology following hemorrhagic shock, and found that PHSML drainage reduced the spleen injury caused by hemorrhagic shock.

It is well known that TLRs mainly lead to two mainly signaling pathways, such as the MyD88-dependent and MyD88-independent pathways (also called TRIF-dependent pathways), to recognize the pathogen-associated patterns or danger-associated patterns. More and more studies reported that TLR plays an important role in inflammation induced by hemorrhagic shock and resuscitation^24^. We found that hemorrhagic shock partly increased the mRNA or protein expressions of the downstream effectors of TLR2/TLR4 signaling pathways, such as MyD88, TRIF, TRAF3 and TRAF6. However, PHSML drainage followed by hemorrhagic shock significantly decreased the mRNA and protein levels of the above molecules. It is suggested that PHSML drainage remarkably reduces the splenic inflammatory response, which is involved in the beneficial effect of PHSML drainage, alleviating spleen injury following hemorrhagic shock.

TIPE2 is a research hot spot and important member of the TIPE family, and is a negative regulator in the process of innate immune response. TIPE2 is a critical regulator of T cell receptor (TCR) and TLR signaling^17^. Its structure includes highly conserved TIPE2 homologous TH (TIPE homology) domain, which is composed of 7 α helix^25^ ^,^ ^26^. It is reported that hepatitis C virus (HCV) could inhibit the expression of TIPE2 to enhance the TLR signaling pathway to promote the occurrence of chronic hepatitis in chronic hepatitis C infection^27^. In addition, the overexpression of TIPE2 in macrophages can play a negative role in innate immunity by inhibiting TLR signal transduction in arthritis^28^. However, it is not clear how hemorrhagic shock affects the expression of TIPE2 and its effect on splenic tissue injury. Our results showed that hemorrhagic shock increased the protein expression of TIPE2, which was reversed by PHSML drainage. The results indicated that PHSML drainage decreased excessive anti-inflammatory response, thereby maintaining the inflammatory response balance. But its detailed molecular mechanism still remains unclear and needs to be further studied. Thus, in the future, we will use TLR2^-/-^ and TLR4^-/-^ mice to further explore the interaction between TIPE2 with the downstream effectors of TLR2/TLR4 signaling pathway, such as MyD88, TRIF, TRAF3 and TRAF6.

## Conclusion

These current results indicate that PHSML drainage alleviates hemorrhagic shock-induced spleen injury and reduces the expressions of TIPE2, MyD88, TRIF, TRAF3 and TRAF6.
